# From codes to language: is the ICF a classification system or a dictionary?

**DOI:** 10.1186/1471-2458-11-S4-S2

**Published:** 2011-05-31

**Authors:** Luigi Tesio

**Affiliations:** 1 Department of Human Physiology and Chair of Physical and Rehabilitation Medicine, Università degli Studi, Milan, Italy; 2 Department of Neurorehabilitation Sciences, Istituto Auxologico Italiano, IRCCS, Milan, Italy

## Monitoring disability across the world: is the ICF the answer?

In a recent international seminar held in Rome [1], an experts’ meeting explored the suitability of the International Classification of Functioning, Disability, and Health (ICF, [2]) as a tool to implement the Convention on the Rights of Persons with Disabilities [3] passed by the United Nations General Assembly in 2005, and now being an instrument of international law valid in many States across the world. The reader of this issue of BMC Public Health has the unique opportunity to get an overview of successful applications of ICF, but also of emerging concerns and difficulties. The ICF was introduced in 2001. Its history dates back to its progenitor, the International Classification of Impairments, Disabilities, and Handicaps, published in 1980 [4]. The ICDH conceptual framework was quite revolutionary: the “consequences of the disease” at organ, person, and person-community levels were given an official conceptualization (impairments, disabilities, and handicaps, respectively), and were coded according to a taxonomy independent of the old established taxonomy of diseases issued by the World Health Organization (International Classification of Diseases, ICD). “Symptoms” like “”difficulty walking” became a condition worth coding ( and thus, studying and treating) “per se”. “Phenomena” were upgraded to “reality” rather than being underestimated as “appearance” [5]. Rehabilitation became an autonomous form of medical care at any stage of the disease or the disablement process, and thus a respected Specialty: it was no more bound to a palliation coming after “true” care became ineffective. The new ICF model emphasized the value of the individual from a societal perspective: “disability” was up-coded (actually, sidelined) to a generic “umbrella term”, under which a positive gradient towards “enablement” was placed. Activity replaced disability, and participation replaced handicap. Whatever a disabled person can achieve “in the context of health experience” is now better than nothing, rather than being less than an ideal standard. The bidirectional flow from organ impairment to person’s performance, to his/her social participation actually became a 3D space expanding along two more axes, through the interactions with individual diseases and individual living environments, respectively (see ref. [6], Fig.1). “Limitations” and “restrictions” were severed from the “intrinsic” person’s status and were ascribed to the community context. Personal bad luck was obscured, and responsibilities of policy makers were spotlighted.

Yet, something went wrong with this otherwise successful project: the philosophic and ethical construct gained an enthusiastic consensus, while the coding structure of the model is still awaiting for wide acceptance and routine application across the health care world [7].

Specialists in Physical Medicine and Rehabilitation (I am one of them) might be considered biased towards a medically oriented view of disability. On the other hand, bio-medicine considers us, the physiatrists, too much biased towards a social view of diseases [8,9]. This entitles me to express some opinions and comments while claiming for a decent neutrality.

## International experiences: successes and concerns

The successes emerging from the set of articles are well represented by the paper by Kostanjsek, a WHO officer [6]. There have been plenty of applications of the ICF model and coding system in fields like legislation, health care planning, disability surveys and policy monitoring. The author reminds that the ICF ancestor, the ICIDH, “was substantially ignored by disability data users”. This positive attitude pervades three more papers, by Madans et al.[10], Good [11] and Madden [12], respectively. The former reports on the experience of the Washington Group on Disability Statistics, a voluntary working group made up of representatives of over 100 National Statistical Offices. Their goal is attaining a very practical ICF-based instrument allowing to measure disability as a fundamental component of the monitoring of the UN convention across the whole world, despite the cultural, linguistic and metric challenges raised by this ambitious undertaking. Good’s paper [11] reports on the comparison of the results of the pilot Irish National Disability Survey held in 2006, which included the ICF coding, and the non-ICF based Census held in 2002 and 2006. The former proved to be much more sensitive than the latter in detecting disabling conditions, also discounting for the methodological differences across the surveys, and it provided explicit knowledge about environmental barriers. The paper by Madden [12] outlines the Australian disability system, focusing on the services for specialist disability and for income support. The classifications in force of “impairments” as a “permanent” condition and as a mixed activity/participation concept, and of “job capacities” without any consideration for environmental factors, appear very far from what the UN conventions is mandating. An attempt is made to translate the present codes into ICF codes. The authors provide examples that this is possible, yet little applicable to administrative decisions on individuals, given the mixed and/or blurred content of the former, compared to the sharper ICF construct. By contrast, the application of the pure ICF coding led to an unprecedented sensitivity in evidencing both disability (e.g. a two-fold rate of disability was detected across adult Indigenous people, compared to other Australians) and the related unmet needs. The paper by Hollenweger [13] stands perhaps midway down a gradient of satisfaction. This article sheds light on the intersection between children disability and the provision of educational services in Switzerland. How much “special” must be these services? Defining “eligibility” to “special services” should aim at fostering inclusiveness and participation, thank to tailored interventions, not segregation: yet, the profile of individual needs “should not be blurred just to affirm general principles of equity”. The ICF coding was asked to walk this tightrope and it has been recently implemented in the “eligibility” decision-making process. Seen from the educational perspective, the participation edge of the ICF should be highlighted. Even more, the estimated potential for participation level in the adult phase, beyond the assessment of the present level, should widen the whole ICF model for better decisions on eligibility.

Still within an optimistic view of the ICF, pragmatic concerns on the implementation of the UN convention arise from the work of Bickenbach [14]. After a thorough survey of the basic features of the convention, he pinpoints how wide are the goals (and the many devisable targets) brought to the fore and how much ill- (if not un-) defined are the proposed monitoring processes: a potential cause for generic “monitoring” within each Country, and inhomogeneity across Countries. The ICF, bridging the person-environment interface, appears as a promising link between coding of wide social goals facilitating political consensus, and technical measurement of focal targets, represented by individual properties and needs.

More severe concerns on the ICF itself are raised by Salvador-Carulla [15]. The ICF properties as a classification system are debatable, given that “major challenges of ICF as a taxonomy remain unsolved”. The “ontology” (let us simplify into “very nature and identity”) of ICF codes is intentionally shaded (or, if you so prefer, multi-potent). For instance, it may well happen that classifying a domain as either activity, participation, or both is left to the user. Problems arise also when the ICF is used as a reference framework for health-related functioning. For instance, the link between health conditions and impairments is much tighter than that between activity and participation. This makes the ICF a useful framework (and perhaps a measure system?) for most health care models based on independence in daily living that focus on mobility, but not for models taking into account psychiatric impairments and/or pivoting around quality of life, still a controversial concept itself [16] which sees the whole health domain as one component of well-being. The proliferation of “core-sets” of ICF items witnesses the intense search of a firmer “ontologic” anchoring. A paper by Di Nubila et al. [17] reminds us how urgent is the need for solving the problems still raised by the assessment of disability. The authors outline the situation of Brazil, where the classification system in force is still based on “addition of categories based on diseases and sequels” within a purely medical model. A national working group was established in 2007 by the President, in order to “evaluate the model of classification and valuation of disabilities used in Brazil …”. The paper summarises the agenda elaborated so far: a daunting challenge indeed. The ICF appears as a promising conceptual framework, but how to translate it into a system of individual decisions is far from being clear, at the moment.

The prevalent feeling that the ICF is not “combat ready” yet in the arena of health care financing is tempered by a paper by Francescutti et al.[18]. The authors present a concrete realisation of an ICF-based classification system suitable for political decision. They ran a survey of 1051 persons from various Italian regions and representing different conditions of functioning and ages. The goal was building a classification tree allowing allocation of individuals to 6 mutually exclusive classes of general needs for assistance, ranging from pure monitoring to extensive redesigning of facilitators and removing barriers. The intersections between ICF codes of activity and participation, and facilitators and barriers were thoroughly scoured. Through a sophisticated statistical design, they came to a manageable “tree” with just 6 terminal, sensible nodes. Albeit very preliminary, this is an encouraging evidence that ICF coding can lead to practical instruments, bridging the gap between medical/individual and political/community perspectives.

## Getting the global picture

All of the papers emphasise the capacity of the ICF to link the description of disability at individual and societal level. The “impairment” edge, the most “intrinsic” to the individual and the most prone to bio-medical interventions, seems the most reluctant to be merged. In any case, at the moment the system appears as the unique conceptual framework providing codes and numbers allowing policy makers to bridge an otherwise insurmountable gap. Is this bridge really walkable? The link was kept intentionally loose between impairments and participation. The latter domain allows perhaps to code what a population requires of politicians, but only the former domain can code what the individual exactly needs from his/her care providers. Blind elderly, deaf children, stroke and paraplegic adults and psychiatric patients, to name just a few, all share forms of general social needs (e.g. a dedicated legislation), yet they do not require the same forms of rehabilitation (to say nothing, obviously, of biomedical care). Also, a large class of disabled people is not very well outlined by the ICF model, i.e. the one comprising people suffering from disability fed by a chronic disease. This establishes a lifelong vicious circle that I would define as interactive disease/disability condition (IDDC). To cite but a few examples of IDDC, let us consider multiple sclerosis, rheumatoid arthritis, neuromuscular diseases, chronic respiratory and/or heart failure and the like. Care planning through the ICF looks even more troublesome in these cases, given that the disease side should be incorporated.

A second point of concern arises from the unsolved issue of the use of ICF codes as quantitative indicators: the metric properties of the “qualifiers” (actually, ordinal, semi-quantitative thresholds aligned along a less-to-more gradient) are far from being validated. “How much” facilitation is obtained by a “moderate facilitator”? A third point is the system complexity, imposing an exhaustive search for consensus and metric validation of “core sets” of items, out of the over 1400 available, applicable to the most various conditions [19,20].

## A shared origin beneath multiple concerns

I glimpse a common source to all of these problems, namely the unsolved distinction between a rigid classification system and a versatile glossary: in short, the “ontologic-taxonomic” problem. An ideal classification system is made by mutually exclusive codes: what a code is not matters not less than what a code is. “Classes” can come out of various combinations of codes and/or cut-off measures: they must remain mutually exclusive, however [21]. The dominating concern, among the ICF supporters, was the search for comprehensiveness, with some emphasis on the de-medicalization of disability. If “a classification must be exhaustive” [19], then you need a complex architecture of the model and a wealth of codes to cover the largest possible combinations of events. However, this is true for a dictionary as well. The point is that if codes can undergo virtually infinite combinations, then you get a language, not a classification system. The description of individual cases is a sentence: using the words in the dictionary may make the description more communicable, yet the information contents (heavily depending on grammar and syntax as well) remain a subjective choice. Core sets appear as a way to standardise sentences: this is like building an invented language, based on a shared lexicon (the codes) and syntactic and semantic conventions. Invented universal languages are a very old human myth: nonetheless, they never succeeded, despite some popularity (see the examples of Esperanto and the Star Trek’s intergalactic “klingon”). Humans still prefer speaking more than 7000 distinct languages. It is still debated whether their grammar generating rules stem from universal, hard-wired brain circuitries determined genetically or whether brain circuitries are genetically plastic in response to any changing cultural influences [22]. Whatever the answer, languages are dynamic components of distinct human cultures; cultural diversity is a distinct tract of human evolution [23]. This notwithstanding, codes can be very much stable and “universal”: Arabic numerals and the ICD systems (encompassing about 10 000 codes of disease) are not facing the difficulties encountered by the ICF “language”, simply because they are “purer” classification systems. “Ten” means not “nine”, so that “10” is not “9”. If I want to mean “twenty”, I cannot choose the symbols I prefer, although I have infinite options to communicate that I was happier when I was 20 years old. This is not to say that consensus on codes can be overlooked: we can use decimal or binary coding systems, and decide whether or not an infectious disease needs to receive a specific code. Nevertheless, consensus is much more easily reached on words than on sentences: the latter must adapt tumultuously to individual and unpredictable situations. If I want to mean that “people with a given motor impairments need some given architectural barriers to be removed”, the collection of ICF codes is unavoidably arbitrary. Any “core set” should be no more than a check-list, a short and conventional identikit, but researchers are tempted to upgrade them to a scale of “mobility” or “dependence”. Building valid scales requires compliance with the axioms of “fundamental measurement” [24] from the early stages of item selection. Items may well be interchangeable, also across “domains” (e.g. dependence and performance [25], or pain and mobility [26]) provided that they are proved to be homogeneous with respect to a construct defined a priori. In this case, items renounce their “ontology” (bestowed to the construct) and become quantitative ticks along a shared ruler. For instance, “entering a tub” may represent the same level of mobility depicted by “entering a car” [27], yet only the latter item would fit a scale of needs for special transports.

## Suggestions from the field

Possibly, in parallel with the mainstream of research on core sets and on implementation of the ICF in health and social care systems, the ICF should be also thought of as an invaluable universal item bank, rather than a pure classification system lending itself to infinite sub-classifications made by piling up its items. Consensus should be reached first on the constructs to be tackled in any given situation needing intervention (dependence? employability? education level? mobility? poverty? depression?). For impairments, activity limitations and participation restrictions, either classification or measurement might then aim at sharper targets and benefit from a consolidated statistical tradition, thus progressing more safely along their related, yet distinct roads.
